# The predictive value of cortisol in psychodynamic psychotherapy for social anxiety disorder: Extended results of the SOPHONET-Study

**DOI:** 10.1038/s41398-024-02882-3

**Published:** 2024-04-11

**Authors:** Ileana Schmalbach, Michael Witthöft, Bernhard Strauß, Peter Joraschky, Katja Petrowski

**Affiliations:** 1 Department of Medical Psychology and Medical Sociology, University Medical Center of the Johannes Gutenberg-University, Mainz, Germany; 2 Department of Clinical Psychology, Psychotherapy, and Experimental Psychopathology, Johannes Gutenberg-University Mainz, Mainz, Germany; 3 Institute for Psychosocial Medicine, Psychotherapy & Psychooncology, University Hospital Jena, Jena, Germany; 4 University Medical Center Carl Gustav Carus, Technische Universität Dresden, Dresden, Germany; 5 Dresden University of Technology, Carl Gustav Carus Medical Faculty, Department of General Medicine/MK3, Dresden, Germany

**Keywords:** Predictive markers, Human behaviour, Psychiatric disorders

## Abstract

Psychotherapy is an effective treatment for anxiety disorders (AD), yet a vast majority of patients do not respond to therapy, necessitating the identification of predictors to enhance outcomes. Several studies have explored the relationship between stress response and treatment outcome, as a potential treatment mechanism. However, the latter remains under-researched in patients with social anxiety disorder (SAD). We studied *N* = 29 patients undergoing psychodynamic psychotherapy (PDT) within the SOPHONET-Study. Stress reactivity (i.e., area under the curve with respect to the increase; AUCi) was induced by a standardized psychosocial stressor (Trier Social Stress Test; TSST) and assessed by means of adrenocorticotropic hormone (ACTH), blood and salivary cortisol samples before (t_1_) treatment. Samples of these biomarkers were taken −1 min prior stress exposure and six more blood samples were collected post-TSST ( + 1, + 10, + 20, + 30, + 45, + 60 min.). The participants were diagnosed with SAD based on the Structured Clinical Interview for DSM-IV (SCID) and completed the Liebowitz Social Anxiety Scale as well as the Beck Depression Inventory before (t_1_) and after psychotherapy (t_2_). Pre-treatment stress reactivity significantly predicted changes in depression (salivary *p* < 0.001 and blood cortisol *p* = 0.001), as well as in avoidance behavior (blood cortisol *p* = 0.001). None of the biomarkers revealed significant results in fear or in the total LSAS-scores, except for ACTH with a trend finding (*p* = 0.06). Regarding therapy success, symptoms of social anxiety (*p* = 0.005) and depression (*p* < 0.001) were significantly reduced from pre (t_1_) to post-treatment (t_2_). Our study showed that stress reactivity pre-treatment may serve as a predictor of psychotherapy outcome. In this regard, alterations in stress response relate to changes in symptoms of social anxiety and depression after PDT. This implies that patients with chronic stress might benefit from a targeted interventions during psychotherapy, especially to manage fear in social contexts.

## Background

Social connection holds profound implications in several aspects of functioning. On the one hand, strong social bonds are associated with positive health outcomes [1–3]. On the other hand, the consequences of inadequate social connections extend from diminished quality of life [4, 5], poor performance [6, 7] to an increased risk of premature mortality, comparable to the impact of smoking and obesity [1, 8]. Accordingly, leading to high socioeconomic costs [9]. Notably, individuals with Social Anxiety Disorder (SAD) may be susceptible to health risks due to the extreme challenges they face in social interactions. SAD as one of the most common mental disorders (lifetime prevalence 12.1 – 6.6%; [10, 11], is characterized by dysfunctional believes about one-self and others, safety and avoidant behaviors, as well as psychovegetative symptoms (e.g. increased heart rate, flushing, sweating; [12, 13]) during social encounters. These symptoms activate the hypothalamic-pituitary-adrenal axis (HPA), potentially leading to chronic stress [14]. The HPA regulates the stress response by releasing cortisol. For this purpose, the corticotropin releasing hormone (CRH) activates the adrenocorticotropic hormone (ACTH) to stimulates the adrenal glands for cortisol secretion [15]. Its release is managed by a negative feedback loop to keep balance in the endocrine system (e.g., ACTH secretion is inhibited in case of elevated cortisol levels [16]). Chronic activation of the stress axis results in an inadequate HPA functioning (e.g., hyper- or hyporesponsiveness [17, 18]), which is considered a predisposing factor for both, mental and somatic disorders [18, 19]. Several studies emphasize the crucial role of HPA-functioning in fear modulation and extinction for therapy success [20–23]. Therefore, interventions aiming a balanced stress response are paramount. The aforementioned evidence underlines the relevance of stress regulation in the treatment of anxiety-related disorders. However, findings on the relationship between stress reactivity and psychotherapy outcome are mixed and in SAD under-researched. On the one hand, patients with the lowest cortisol stress response to an acute stressor (i. e., math task, flooding) prior treatment profited least from psychotherapy (CBT; [24–26]). On the other hand, Wichmann et al. [27] revealed that patients with *higher* (blood) cortisol reactivity (DEX-CRH-test) exhibited the least improvement by psychotherapy. Conversely, further research suggested that cortisol reactivity prior treatment was not related to psychotherapy outcome [28, 29]. Overall, it is unclear whether stress reactivity prior treatment is related to psychotherapy outcome. Nonetheless, the observed heterogeneity in the aforementioned outcomes may be partly attributed to differences in the studied population (e.g., anxiety disorders in general vs. disorder specific samples), methods of stress induction, assessed biomarkers (e.g. ACTH, blood or salivary cortisol) and their handling (e.g., controlling for circadian and ultradian activity) as well as in psychometric assessment (e.g., ceiling effects due to anticipatory anxiety before stress exposure [30]. Furthermore, despite the effectiveness of psychotherapy (i.e., PDT, CBT) for anxiety disorders (AD; [31]) mechanisms of treatment remain unclear. In this regard, non-responders (40%–48%; [31–33]) and high drop-out rates are still problematic [34–36]. Therefore, the identification of predictors that may optimize treatment outcomes is crucial for tailoring interventions enhancing alleviation of anxiety symptoms. Considering the inconsistency of findings and the lack predictive biomarkers in PDT specific to SAD, the aim of the current study is to provide first evidence in this context and extend the results of the SOPHO-NET study [31]. Hereby, the primary aim is to assess the relationship between stress reactivity and psychotherapy outcome. Additionally, in order to overcome past methodological challenges, we aim to examine the hormonal cascade (ACTH, blood and salivary cortisol) prior psychotherapy treatment under highly standardized conditions, i.e., the use of Trier Social Stress Test (TSST; [37]) controlled biomarker handling, and highly trained psychotherapist.

### Hypothesis (H_1_)

Based on previous findings we hypothesized that cortisol reactivity (i.e., AUCi) significantly predicts therapeutic outcome in terms of psychological symptoms of depression and social anxiety. Hereby, we expect a significant negative relationship between cortisol reactivity and change in symptom severity (i.e., anxiety and depression). The latter implies that alterations in the stress response relate to an improvement in symptoms of anxiety and depression. That is, patients with hyporeactivity benefit less from treatment, while patients with a significant cortisol increase are expected to show a significant symptom decrease pre (t_1_) vs. post (t_2_).

## Method

### Procedure

The SOPHO-NET –Research Network on Psychotherapy for Social Phobia is one of five research networks on psychotherapy comprising several interrelated studies focusing on SAD. One of the SOPHO-NET studies [31, 38] investigated the efficacy of the PDT and CBT in the context of a multicenter randomized controlled trial of *N* = 495 patients with SAD. As part of the SOPHO-NET project, outpatients were recruited and randomly assigned to manual-guided psychotherapy or a waiting list condition. PDT as well as CBT were significantly superior to waiting list for both, remission and response. Primary outcome measures were rates of remission and response, according to the Liebowitz Social Anxiety Scale (LSAS) applied by raters blind to group assignment. Several secondary measures including the BDI were implemented as well. For the purpose of the present study, we only analyzed PDT outcomes related to symptoms of depression and social anxiety. Psychological symptoms were collected before and after treatment. The study protocol received approval from Ethics Committee of the Medical Faculty of the University of Göttingen and Dresden (Ethics-Nr. No#EK7012006).

### Psychotherapy

Our participants received up to 25 individual 50-minute treatment sessions weekly. The PDT-therapy sessions applied were delivered based on Luborsky’s manual specially adapted for SAD [39, 40]. The later encompasses supportive and expressive interventions (e.g., establishing a secure helping alliance, addressing patient’s underlying core conflictual relationship-patterns). All of the study psychotherapists had completed an additional psychotherapeutic training or were advanced and received regular supervision. For quality purposes, all the treatment sessions were recorded after informed consent. An extended description of the implementation and procedures to ensure treatment fidelity and integrity is described elsewhere [31].

### Laboratory stress induction

In order to increase the validity and quality of the results, we collected ACTH, blood and salivary samples as proxies of the HPA activity. For the purpose of minimizing circadian variations in cortisol levels, the study participants were consecutively scheduled for the TSST between 3:00 and 6:00 p.m. The participants were solicited to avoid comestibles and smoking for at least two hours prior and during laboratory testing. TSST exposure followed a baseline period of 25 min. Before exposure, the participants were informed that they will be recorded. This experimental paradigm reliably activates the HPA [41] combining uncontrollability, social evaluation, as well as arithmetic tasks in a time frame of 15 min. At first, the participants are invited to prepare for a job interview. Next, the interview takes place by the TSST-committee in lab coats. Lastly, the arithmetic tasks are conducted (e.g., subtracting in steps of 17). A detailed description of our protocol is available by Kirschbaum et al. [37].

#### Cortisol collection

One sample (−1 min) was taken before TSST exposure. Immediately after TSST completion, six more samples (+1 min, +10 min, +20 min, +30 min, +45 min, +60 min) were extracted at regular intervals. In total, seven samples were acquired and taken in a supine body position by trained staff members.

### Methods of cortisol collection and analysis

Salivary samples were collected by means of Salivette swabs (Sarstedt, Nümbrecht, Germany). The sampling consisted in the intra-orally moistening of a cotton roll for 1 min. prior placement into a salivette swab. Before analysis, the samples were centrifuged at 3000 rpm for 5 min. to produce a clear supernatant of low viscosity. 50 μl were removed for cortisol analysis using a commercially available immunoassay with chemiluminescence detection. The lower detection limit of this assay is 0.43 nmol/l. Intra- and inter-assay coefficients of variation were below 8% for low (3 nmol/l) and high (25 nmol/l) cortisol levels, respectively. Concerning the collection of blood and ACTH samples, blood was extracted by means of an intravenous cannular after 45 min. of rest at the laboratory. The blood samples resided into Serum-Gel-Monovette® (Sarstedt, Nümbrecht, Germany) tubes. Directly after collection, the samples were centrifuged for 10 min at 4 °C and 3000 rpm. Following aliquoting, the samples were stored at −80 °C and at −20 °C freezer before being assayed.

### Participants

The participants of the study at hand were recruited between April, 2007 to April, 2009 at the Psychotherapy Clinic for Psychotherapy and Psychosomatic Medicine of the Technical University Dresden, as part of a larger sample within the SOPHO-NET study. In said clinic *N* = 29 patients gave consent to take part in the TSST and also finalized the psychotherapy. The pre and post LSAS measurement of six participants as well as the pre and post-measurement of the BDI of four participants was not available due to non-matching codes. The inclusion criteria consisted in participants between 18 and 70 years, a diagnosed SAD based on the Structured Clinical Interview for DSM-IV (SCID; [42])and finally a score above 30 on the LSAS (German version, [43, 44]. Participants with the following criteria were excluded: psychotic and acute substance-related disorders, cluster A and B personality disorders, prominent risk of self-harm, organic mental disorders, severe medical conditions, and concurrent psychotherapeutic or psychopharmacological treatments.

### Demographic and clinical characteristics

The characteristics of the participants along with the demographic information of the study sample is displayed in Table 1.

**Table 1:** Sociodemographic data.

	*n*	*%*
Gender		
Male	9	31.0
Female	15	51.7
without	5	17.2
Marital status		
Single	23	79.3
Married	4	13.8
Divorced	2	6.9
Relationship status		
Marriage	5	17.2
Close relationship	7	24.1
Short-term	1	3.4
Long-term	16	55.2
Education		
None	1	3.4
Secondary school	3	10.3
Intermediate secondary school	5	17.2
Vocational Baccalaureate	9	69.0
General Baccalaureate	11	37.9
Vocational qualification		
None	1	3.4
In training	5	17.2
Apprenticeship	10	34.5
Technical college/university	13	44.8
Employment status		
Employed	24	82.8
Unemployed	5	17.2

### Measurement instruments and diagnostics

Trained and independent clinical psychologists conducted the structured clinical interviews for diagnostic purpose by means of the SCID [41]. Based on the structured format, the interviewer asks certain questions and codes the answers in order to make a differential diagnosis. *Liebowitz Social Anxiety Scale* (LSAS; [43, 45]). This scale is a clinician-administered, semi-structured interview for the assessment of SAD-related symptoms. It measures anxiety and avoidance behavior in interaction and performance contexts within 24 items on a four-point Likert-scale (0 = none/never to 3 = strong/almost always). Each item is separately rated, for fear (0 to 3; 0 = none, 3 = severe) and avoidance (0–3; 0 = never, 3 = usually). Its total score encompasses 0–144 points. Additionally, a total score can be determined as an indicator of overall symptom severity. The scale has an internal consistency of α = 0.94 and a retest reliability of *r*_*tt*_ = 0.84.

#### Beck Depression Inventory (BDI; [46, 47])

The BDI is a 21-item self-report questionnaire that evaluates the severity of symptoms of major depression in the current week of administration. Respondents answer in a scale from 0 (e.g., “I am not sad”) to 3 (e.g., “I am so sad or unhappy that I can hardly stand it anymore”). The values of each item are summed and compared to cut-off values for depression: 0–12 (no depression), 13–19 (mild), 20–28 (moderate), and 29–63 (severe). The psychometric properties of the BDI are satisfactory (α = 0.86; α = 0.92; [48]).

### Statistical analyses

The statistical analyses were performed using *R* [49]. The psychometric data was normally distributed according the Kolmogorov-Smirnov test after Lilliefors correction. The cortisol data were not log-transform since the majority of the normality tests were not significant. The stress reactivity was measured by means of area under the curve with respect to the increase (AUCi; [50]). In order to predict the psychotherapy outcome (i.e., pre and post measurements in depression and social anxiety) based on the hormonal response (i.e., ACTH, salivary, and blood cortisol) to the TSST, we calculated a regression analysis with AUC_I_ as an independent variable. In order to find out symptom reduction after psychotherapy, we calculated a *t*-test for paired samples. The internal consistency of the scales was computed and expressed in Cronbach’s Alpha. Participants with pairwise incomplete data were excluded from the analyses (four for BDI, and six for LSAS). There were no significant differences between participants with missing and non-missing data when evaluating the groups using χ²- and *t*-test. The significance level for all the analysis was defined as *p* < 0.05.

## Results

### Reliability

The reliability of the scales is expressed in Cronbach’s Alpha and reported in Table 2.

**Table 2:** Scales statistics and paired comparisons of pre-post measurement of psychological symptoms.

								95% CI				
Scales	*M*	SD	*α*			Δ*M*	ΔSD	Lower	Upper	*t*	df	*p*
LSAS				Pre-post comparisons^+^								
LSAS-Fear pre	39.47	18.38	0.82	Pair 1	LSAS-Fear	6.21	17.22	1.23	13.66	1.73	22	0.049^*^
LSAS-Avoidance pre	36.17	14.17	0.82	Pair 2	LSAS -Avoidance	9.30	13.78	3.34	15.26	3.23	22	0.002^**^
LSAS-total pre	77.39	31.17	0.83	Pair 3	LSAS-total	17.21	28.54	4.85	29.57	2.88	22	0.005^**^
LSAS-Fear Post	33.26	13.45	0.82									
LSAS -Avoidance post	26.87	15.30	0.82									
LSAS-total post	60.17	28.32	0.83									
BDI				Pair 4	BDI	9.84	6.60	6.66	13.02	6.49	18	< 0.001^**^
BDI pre	16.42	7.97	0.86									
BDI post	6.58	7.53	0.87									

*LSAS* Liebowitz Social Anxiety Scale, *BDI* Beck Depression Inventory. *p < 0.05, **p < 0.01. ^+^Pair sample *t*-test

### Missing values

There were no significant differences concerning age (*t*(27) = 1.45, *p* = 0.16) and gender (χ²(1) = 0.34, *p* = 0.56) between participants with missing values vs. participants with complete data sets.

### Predictions

#### Depression (BDI)

**Fig. 1:**
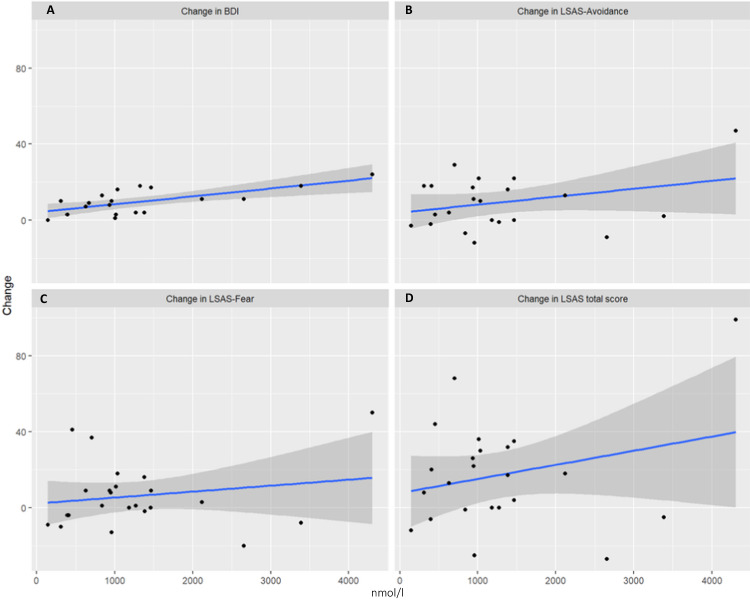
Relationship between stress response (AUCI salivary cortisol) and therapy outcome related to symptoms of depression and social anxiety. **A** Change in BDI; **B** change in LSAS-Avoidance; **C** change in LSAS-Fear; **D** change in LSAS-total score. Raw values of salivary cortisol. LSAS Liebowitz Social Anxiety Scale, BDI Beck Depression Inventory.

**Fig. 2:**
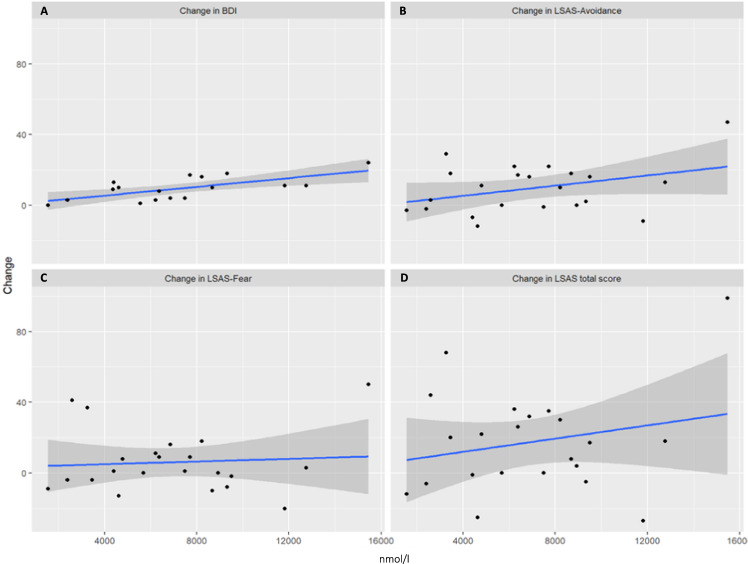
Relationship between stress response (AUCI blood cortisol) and therapy outcome related to symptoms of depression and social anxiety. **A** Change in BDI; **B** change in LSAS-Avoidance; **C** change in LSAS-Fear; **D** change in LSAS-total score. Raw values of salivary cortisol. LSAS Liebowitz Social Anxiety Scale, BDI Beck Depression Inventory.

**Fig. 3:**
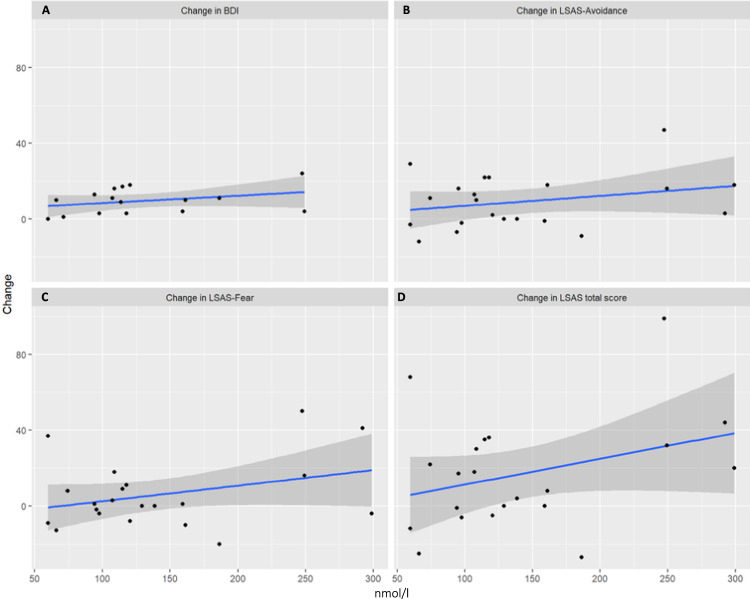
Relationship between stress response (AUCI ACTH) and therapy outcome related to symptoms of depression and social anxiety. **A** Change in BDI; **B** change in LSAS-Avoidance; **C** change in LSAS-Fear; **D** change in LSAS-total score. Raw values of salivary cortisol. LSAS Liebowitz Social Anxiety Scale, BDI Beck Depression Inventory.

In terms of AUCi, salivary cortisol (*β* = 0.67, *t*(17) = 3.70, *p* < 0.001), as well as blood cortisol (*β* = 0.68, *t*(15) = 3.56, *p* = 0.001) significantly and positively predicted changes in depression (see Figs. 1A–2A). It is suggested that a stronger cortisol response is associated with a better treatment response, as manifested in the BDI change score. On the other hand, ACTH (*β* = 0.33, *t*(14) = 1.28, *p* = 0.11) was not predictive (see Fig. 3A).

#### Total Anxiety scores (LSAS)

In terms of AUCi neither salivary (*β* = 0.25, *t*(21), = 1.22, *p* = 0.11) nor blood cortisol (*β* = 0.22, *t*(20) = 1.04, *p* = 0.15) or ACTH (*β* = 0.33, *t*(19) = 1.54, *p* = 0.06) significantly predicted changes in overall symptoms of SAD, but revealed a trend result (see Figs. 1D–3D).

##### LSAS-subscales. avoidance score

In terms of AUCi, blood cortisol (*β* = 0.67, *t*(15) = 3.56, *p* = 0.001) significantly and positively predicted changes in avoidance behavior (see Fig. 1B). Salivary cortisol exhibited a trend result (*β* = 0.30, *t*(21) = 1.44, *p* = 0.08), while ACTH manifested no effect (*F* ≤ 1, *p* ≥ 0.36).

##### Fear score

In terms of AUCi none of the biomarkers revealed significant results (*t* 1, *p* ≥ 0.36).

### Therapy success

#### Pre (t_1_)-vs-post (t_2_) measures

Concerning total anxiety scores, symptoms were significantly reduced from t_1_ to t_2_, (LSAS_tot_ *t*(22) = 2.88, *p* = .005). Symptoms of *fear* and *avoidance* behavior, as well as depression were significantly reduced from t_1_ to t_2_ (see Table 2).

## Discussion

The aim of the present study was to extend the results of the SOPHO-NET study and determine whether the psychobiological stress response operates as a predictor of psychotherapy outcome in SAD. In general, our results suggested that stress reactivity is associated with changes in symptoms of depression and anxiety following PDT. Hereby, cortisol reactivity predicted changes in depression and avoidance behavior, however, not in fear related to social anxiety. Concerning treatment success, symptoms of social anxiety including fear and avoidance as well as depression were significantly reduced by manualized PDT-treatment. In detail, our hypothesis received partial support. With regards to the depressive symptoms, a stronger (salivary and blood) cortisol response was associated with a better treatment response, while ACTH and blood cortisol only demonstrated a trend result. This finding is in accordance with Dielemann et al. [24] demonstrating that (salivary) cortisol stress reactivity predicted depressive symptoms even at one-year follow-up in anxiety disorders. In terms of SAD related symptoms, blood cortisol significantly and positively predicted changes in *avoidance* behavior, while salivary cortisol only exhibited a suggestive tendency in this regard. Interestingly, ACTH was not significantly predictive. A similar tendency was found by Siegmund et al. [25] with no changes in ACTH and by Wichmann et al. [26]. Their study suggested that compared to ACTH, the highest (blood) cortisol reactivity to the TSST prior psychotherapy showed the most improvement in (agoraphobic) avoidance. This outcome can be explained based on hormonal mechanisms, whereby blood and salivary cortisol reflect overall hormonal levels and biological active cortisol. In comparison, ACTH reveals the capacity of the adrenal glands to produce cortisol [51, 52]. In this case, our data showed no relationship between ACTH and therapy outcome, hence the role of the CRH-receptors remains unclear. Even so, this finding should be explored in a follow-up study. In the current study, stress reactivity markers (ACTH, blood and salivary cortisol) did not significantly predict changes in total anxiety scores (LSAS) post-treatment. Only ACTH showed a marginal trend, and there were no notable associations with the LSAS-fear subscale. The aforementioned findings contradict those of Wichmann et al. [27], who found that higher cortisol reactivity was linked to higher disease severity after CBT. At the same time, our latter findings fit the results of Meuret et al. [29] and Fischer and Cleare [28], suggesting no predictive power of (neither blood nor salivary) cortisol reactivity prior psychotherapy for AD. Even so, these outcomes did not illustrate disorder specific aspects of the SAD symptomatology. In this regard, it is known that HPA function across AD has not revealed a consistent pattern of endocrine disturbance [53], explaining some of the inconsistencies. In sum, our study suggested that alterations in stress response are related to improved symptomatology. In this regard, patients with greater stress reactivity profited most from psychotherapy, especially in terms of avoidance behavior. As such, it is implied that the stress response could serve as a mechanism of treatment. Nevertheless, this mechanism was not revealed for total *anxiety scores* and *fear*. Hence, it is conceivable that certain anxiety related behaviors (e.g., avoidance) are more responsive to psychotherapy than others. Hence, the study at hand offers preliminary evidence that cortisol responsivity to stress may act as a potential mechanism of treatment, indicating a possible marker for more personalized models of healthcare (e.g., ideographic treatment planning).

### Limitations

A possible shortcoming of the study at hand refers to the constrained sample size, considering the missing data in pre and post measurements of psychometric data for pairwise comparisons. Consequently, our findings are limited in their generalizability. Regardless, the sample size is comparable to those reported in similar studies [54]. Additionally, the reported results make a strong contribution in the field of PDT based interventions. A strength of this study are the highly controlled conditions, as well as the use of several biomarkers illustrating both, cortisol activity in the periphery as well as in the adrenals. Further, the use of biomarkers strength psychometric outcomes especially in the realm of PDT, since there is a lack of evidence in said regard. For future research it is recommended to test whether adjuvant interventions for stress reduction might help patients also reduce symptoms of depression. Moreover, not only hormonal stress reactivity, but also habituation would be of interest as a therapeutic outcome. Due to the risk that stress vulnerability poses on the organism, not only cortisol and ACTH, but a further control cycle with immune parameters as predictor (e.g., IL-6) should be studied.

## Conclusion

In conclusion, our study showed that stress reactivity (pre-treatment) can be used as a predictor of psychotherapy outcome and that alterations in stress response relate to changes in symptoms of anxiety and depression. An adequate reactivity may indicate a better treatment response in terms of social anxiety. Patients with chronic stress might benefit from a targeted interventions to manage fear and depressive symptoms during psychotherapy.

## Data Availability

The datasets used and/or analyzed during the current study are available from Prof. Katja Petrowski: kpetrows@uni-mainz.de on reasonable request.
